# Pharmacy location and medical need: regional evidence from Canada

**DOI:** 10.1186/s12913-022-08709-5

**Published:** 2022-11-03

**Authors:** Paul Grootendorst

**Affiliations:** Leslie Dan Faculty of Pharmacy, University of Toronto, 144 College St, Toronto, ON M5S3M2 Canada

**Keywords:** Regional distribution, Community pharmacy, Access, Canada, Income, Seniors, Medical need

## Abstract

**Background:**

Pharmacists in Canada are assuming an increasingly important role in the provision of primary care services. This raises questions about access to pharmacy services among those with medical care needs. While there is evidence on proximity of residents of Ontario and Nova Scotia to community pharmacies, there is little evidence for the rest of Canada. I thus measured the availability of pharmacist services, both the number of community pharmacies and their hours of operation, at both the provincial and sub-provincial level in Canada. Next, I measured associations of indicators of medical need and the availability of pharmacist services across sub-provincial units.

**Methods:**

I collected data, for each Forward Sortation Area (FSA), on medical need, measured using the fraction of residents aged 65 + and median household income, and pharmacist service availability (the number of community pharmacies and their hours of operation, divided by the FSA population). Linear regression methods were used to assess associations of FSA-level service availability and medical need.

**Results:**

There are between 2.0 and 3.3 community pharmacies per 10,000 population, depending on the province. There are also provincial variations in the number of hours that pharmacies are open. Quebec pharmacies were open a median of 75 h a week. In Manitoba, pharmacies were open a median of 53 h a week. The per capita number of pharmacies and their total hours of operation at the FSA level tend to be higher in less affluent regions and in which the share of residents is aged 65 or older. Provincial differences in pharmacy availability were still evident after controlling for medical need.

**Conclusion:**

Community pharmacies in Canada tend to locate where indicators of health needs are greatest. The impact on patient health outcomes of these pharmacy locational patterns remains an area for future research.

**Supplementary Information:**

The online version contains supplementary material available at 10.1186/s12913-022-08709-5.

## Background

Pharmacists’ role in the health care systems of different countries has expanded over the last three decades, for several reasons. First, demand for medication dispensing and counselling – core pharmacy services – has grown in lockstep with the variety of prescription medications that are available. Second, pharmacists’ scope of practice has expanded. In Canada and in various other jurisdictions, pharmacists are administering vaccines, screening for hypertension and other diseases, counselling on smoking cessation, prescribing drugs for minor ailments, and providing a variety of other primary care services [1, 2].

For pharmacists to effectively deliver healthcare, they need to be accessible to patients. Patient access to pharmacist services has been the focus of a growing literature. These include studies of pharmacy service availability: across urban and rural areas of New Zealand [3] and the United States; [4] in areas populated predominantly by lower income households in England [5] and in various cities in the United States; [5–8] and among the elderly living in Scotland [9] and Portugal [10]. Waterson developed an economic framework to determine the optimal number of pharmacies and compared the density of pharmacies in Melbourne, Australia to the optimum [11]. Jagadeesan and Wirtz review studies of local pharmacist service availability in other countries [12]. The Organisation for Economic Co-operation and Development (OECD) has compiled data on the pharmacy density of member countries [2].

Several studies have investigated patient access to pharmacy services in Canada. Two focused on access in the provinces of Ontario [13] in 2010 and Nova Scotia [14] in 2011. The studies found that most urban dwellers in these provinces live close to a pharmacy; access is somewhat lower in rural areas. Another study focussed on pharmacy access across neighbourhoods in the Greater Toronto Area in 2014; Toronto is the capital of Ontario [15]. Neighbourhoods were characterized by level of healthcare need, i.e., the capacity to benefit from healthcare. Need was measured using average household income, and the elderly (65 +) and child (0–9) shares of the population. The study found that about 14% of the population had low access relative to healthcare need; about 13% of the population was over-serviced relative to need [15].

Little is known about pharmacy access outside of Ontario and Nova Scotia. This paper, therefore, examined pharmacist services availability across Canada and also examined how pharmacist services availability varied with two indicators of healthcare need that are routinely used in the aforementioned literature. These characteristics are older age (the share of the population 65 +) and median household income.

## Methods

This study used data on pharmacist services availability and population characteristics for each of roughly 1,300 geographic regions, called Forward Sortation Areas (FSAs), that span Canada’s 10 provinces. These data were used to estimate, via ordinary least squares (OLS), the parameters of linear regression models of FSA-level pharmacist services availability as a function of, *inter alia*, the share of the FSA population 65 + and median household income.

### Measuring FSA-level pharmacist services availability

Pharmacist services availability was measured using the number, and usual hours of operation each day of the week, of community pharmacies located in residential FSAs in Canada’s 10 provinces. Pharmacy location data were obtained from an insurance claims adjudicator, Express Scripts International (ESI), which provided addresses of the community pharmacies in each province that billed private drug plans in the period April-June 2019. These lists were verified by comparing with lists obtained by the pharmacy regulatory bodies in each province, except Quebec. I did not have any master pharmacy lists for Quebec so I was unable to verify the ESI data as I did for the other provinces. The Ontario and British Columbia pharmacy regulatory authorities provided pharmacy-specific operating hours. For the remaining provinces, the study team manually determined the usual hours of operation each day of the week of each pharmacy by consulting the pharmacy website. If the website did not provide the information, a member of the study team called the pharmacy. Pharmacies were assigned to FSAs using the pharmacy postal code; the FSA identifier is the first three characters of the postal code [16].

The ESI data exclude “central fill” facilities – pharmacies that prepare large number of prescriptions for distribution to residents of long-term care facilities and other institutions. However, not all of the pharmacies in the ESI data are traditional community pharmacies; some of these pharmacies specialize in mail-order (for both domestic and international patients), and some provide exclusively biologics and other specialty medicines. I removed these types of pharmacies.

### Measuring FSA-level population characteristics

I obtained data on the residential population, by age group, and median total household income for 2015 of each FSA from the 2016 Census of Canada, [16] which Statistics Canada conducted in July 2016. The population data were used to express the number of community pharmacies per FSA and the total number of hours that they are open into per capita terms.

One defect of the Census-based household income variable was that it was measured for 2015, four years prior to the 2019 measurement of pharmacy density. I thus used an alternative measure of income, collected in 2019. This was average total personal income for 2019 declared by tax filers in the FSA; these data were reported by the Canada Revenue Agency [17]. I assessed whether results were robust to this alternative measure of income.

### Selection of FSAs

There were a total of 1,632 FSAs that span the 10 provinces. Some of these FSAs consist mostly of commercial or government buildings and have very small or no residential communities. I removed these by requiring all FSAs to have an area of at least 8 square kilometers and a residential population of at least 100. This left 1,304 FSAs. Of these FSAs, census data were available for 1,282 FSAs [18] and Canada Revenue Agency taxfiler data was available for 1,280 FSAs. The FSA of median size in the analysis sample encompasses an area of 92 km^2^.

### Descriptive statistics

I graphed province-level data on pharmacy availability, including the 10th, 50th and 90th percentiles of normal operating hours, for both the entire week and just weekends, of pharmacies in each province. I also graphed, by province, the fraction of pharmacies open on Saturdays and on Sundays. Finally, I graphed the number of community pharmacies per 10,000 population. I used Statistics Canada estimates of provincial populations for the second quarter of 2019 [17]. (These population estimates were different than the FSA-level population data, which were available for 2016 only.)

I further plotted data on pharmacy availability at the FSA level (the number of pharmacies and total weekly pharmacy operating hours, both per 10,000 population) against median total household income for residential FSA regions across the 10 provinces of the FSA. A lowess curve (a local average of the y-axis variable evaluated at each income value) was superimposed in each of the plots.

### Modelling FSA pharmacy availability

I specified linear regression models of the number of pharmacies, the total weekly pharmacy operating hours, and total weekend operating hours per FSA, all expressed per 10,000 population. These outcome variables were posited to depend *inter alia* on the fraction of the residential population that is 65 years or older and median household income. To allow for non-linearities in the relationship between income and pharmacy availability, I used indicators for 9 of the 10 deciles of median household income. The first decile (the 10% of FSAs with the lowest median household income) formed the reference group. To allow for non-linearities in the relationship between the elderly share of the FSA population and pharmacy availability, I used three indicators of the quartiles of the elderly share of population; the first quartile formed the reference group.

The regression model also includes a variable that indicates whether the FSA is rural or non-rural. (Rural regions are identified by a “0” in the second character of the FSA identifier.) Finally, the model included indicators for each of the provinces. Ontario is the reference group [17]. The Stata program “coefplot” [19] was used to graph regression parameter estimates. To account for possible heteroskedastic errors, the heteroskedasticity-consistent covariance matrix estimator was used.

One defect with OLS regression is that estimates can be sensitive to outlier values of the pharmacy accessibility outcome variables (either the number of pharmacies per capita or the total operating hours per capita). To assess the robustness of the OLS estimates, the models were re-estimated using quantile regression. Specifically, I estimated the conditional median of the outcome variables.

## Results

### Province-level results

**Fig. 1:**
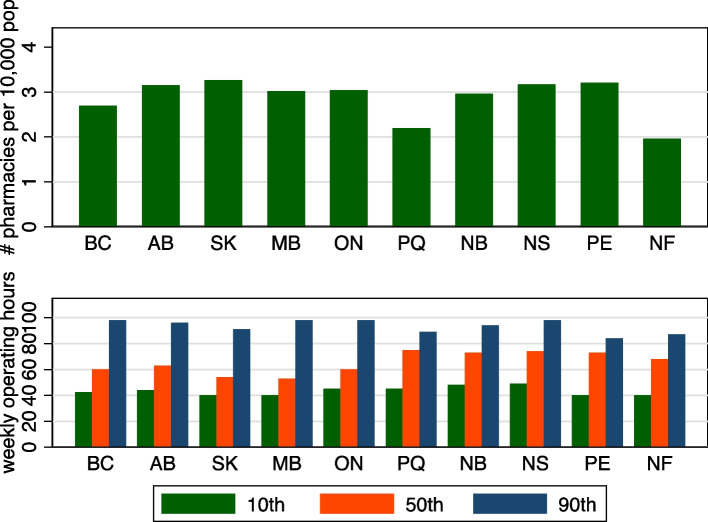
Number of community pharmacies per 10,000 population, by province (top panel). 10^th^, 50th and 90^th^ percentile of pharmacy weekly operating hours, by province (bottom panel). Legend: BC = British Columbia, AB = Alberta, SK = Saskatchewan, MB = Manitoba, ON = Ontario, PQ = Quebec, NB = New Brunswick, NS = Nova Scotia, PE = Prince Edward Island, NF = Newfoundland and Labrador

**Fig. 2:**
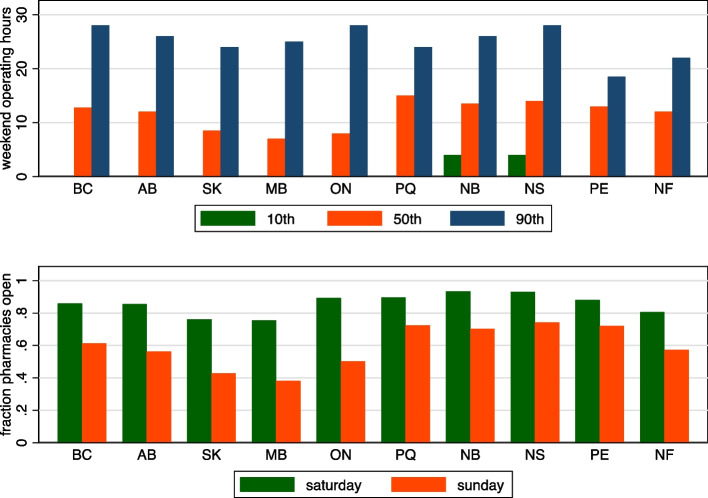
10^th^, 50^th^ and 90^th^ percentile of pharmacy level weekend operating hours, by province (top panel). Fraction of pharmacies open Saturdays and on Sundays, by province (bottom panel). Legend: BC = British Columbia, AB = Alberta, SK = Saskatchewan, MB = Manitoba, ON = Ontario, PQ = Quebec, NB = New Brunswick, NS = Nova Scotia, PE = Prince Edward Island, NF = Newfoundland and Labrador

I first examine graphs of the measures of pharmacy accessibility in each province, presented in Figs. 1 and 2. For completeness, the data are also reported in Tables 1 and 2. The top panel in Fig. 1 shows the provincial variation in pharmacy density. This density varies from 2.0 per 10,000 population in Newfoundland to 3.3 per 10,000 population in Saskatchewan. Accessibility, however, depends not only on the number of pharmacies but also the hours each pharmacy is open. The bottom panel of Fig. 1 shows percentiles of the distribution of pharmacy level normal weekly operating hours. There is little cross-province variation in the 10^th^ percentile of weekly operating hours. However, there is marked variation across provinces in the median operating hours per week – from a low of 53 h in Manitoba to a high of 75 in Quebec. While the median hours per pharmacy is lowest in Manitoba, the 90^th^ percentile is among the highest. This indicates that there is a contingent of Manitoba pharmacies that are open for as long as pharmacies operating in other provinces.

**Table 1:**
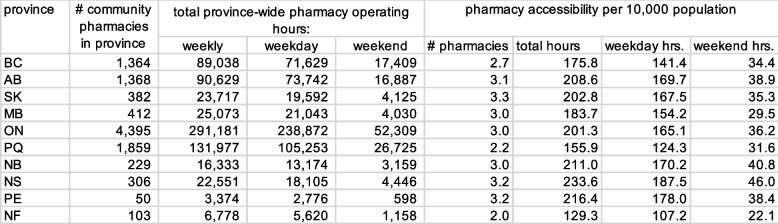
Measures of community pharmacy availability and access, by province, second quarter 2019

**Table 2:**
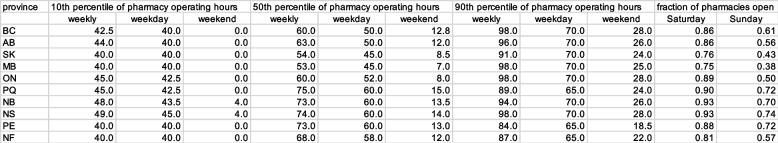
Measures of community pharmacy availability and access, by province, second quarter 2019

Figure 2 focuses on pharmacy accessibility during weekends. The top panel shows the percentiles of the distribution of pharmacy level weekend operating hours. Again, there is marked interprovincial variation in median hours, with the lowest values observed in Manitoba and Ontario and the highest values observed in Quebec. There is less variation in the 10^th^ and 90^th^ percentile of pharmacy weekend operating hours. The bottom panel of Fig. 2 shows the fraction of pharmacies open on Saturdays and on Sundays. The fraction of pharmacies open on these days is the highest in Quebec and the Atlantic provinces.

### FSA-level results

**Fig. 3:**
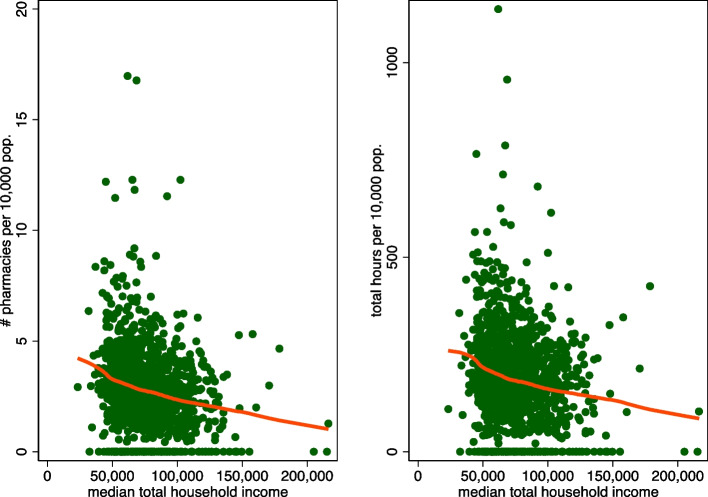
Number of pharmacies per 10,000 population (left panel), total weekly pharmacy operating hours (right panel) and median FSA total household income, in Canadian dollars. Note: the orange lines represent local averages of the y-axis variable for different values of median total household income. Sample size is 1,282 FSAs

I next turn to the analyses of pharmacy accessibility at the FSA level, beginning with Fig. 3, which plots pharmacy density and FSA income (left hand side) and the total number of pharmacy operating hours per week per 10,000 population and FSA income (right hand side). The plots reveal that pharmacy service availability increases as regional median household income decreases. There is, however, substantial variation in pharmacy density and hours for a given level of household income.

Descriptive statistics are presented in Table 3. The median FSA-level household income ranged from $23,317 to $216,260. The average was $75,391 and the median was $71,058. The age 65 + share of the FSA population varied from 0 to 44% and had an average value of 18%. The FSA land area varied from 8 km^2^ to 483,227 km^2^; the median was 92 km^2^. The mean of the province indicators reflect the fraction of FSAs in each province; 31% of the FSAs are from Ontario.

**Table 3:**
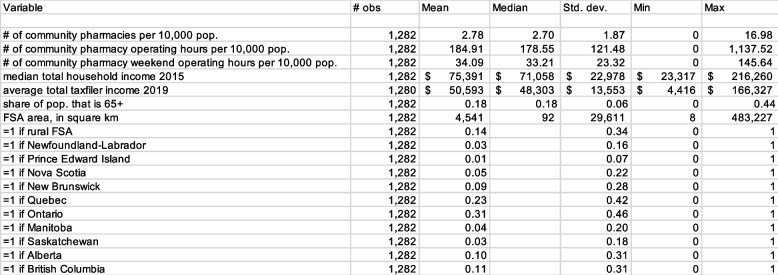
Descriptive statistics

**Fig. 4:**
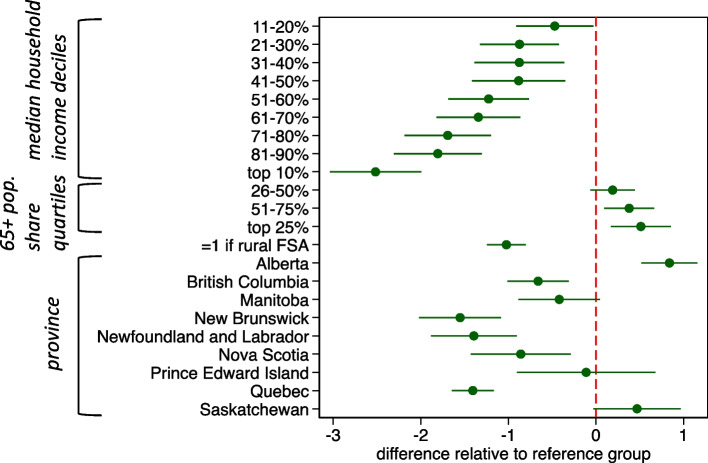
Linear regression of number of community pharmacies per 10,000 FSA population. Note: estimates are the difference in number of community pharmacies per 10,000 population relative to reference group: non-rural Forward Sortation Areas in Ontario, with lowest 10% of median total household income and lowest 25% of elderly population share. Estimated 95% confidence intervals in green

Next, I report estimates of the linear regression models for each of the three measures of pharmacy availability in the FSA, namely the number of pharmacies, the total weekly operating hours and the number of weekend operating hours, all expressed per 10,000 population. Numerical parameter estimates for each linear regression model are reported in Appendix 1. The parameter estimates (and associated 95% confidence intervals) for the model of the number of pharmacies are illustrated in Fig. 4. The estimates reveal the following: First, holding constant other factors, pharmacy density is higher, the lower is the regional median household income. Indeed, there are about 2.5 fewer pharmacies per 10,000 population in the most affluent regions compared to the least affluent regions. This corroborates the results of Fig. 3. Second, there are more pharmacies per capita in regions with a larger elderly population share. Third, pharmacy density is lower in rural regions (about 1 fewer pharmacy per 10,000 population). Finally, pharmacy density also varies across the provinces, even after controlling for household income, demographics and rurality. There are fewer pharmacies per capita in Newfoundland, New Brunswick and Quebec, than in Ontario and more in Alberta. Results are qualitatively similar if the 2019 taxfiler reported income data are used instead of the 2015 household income data reported in the Census (see Appendix 1 for estimates).

**Fig. 5:**
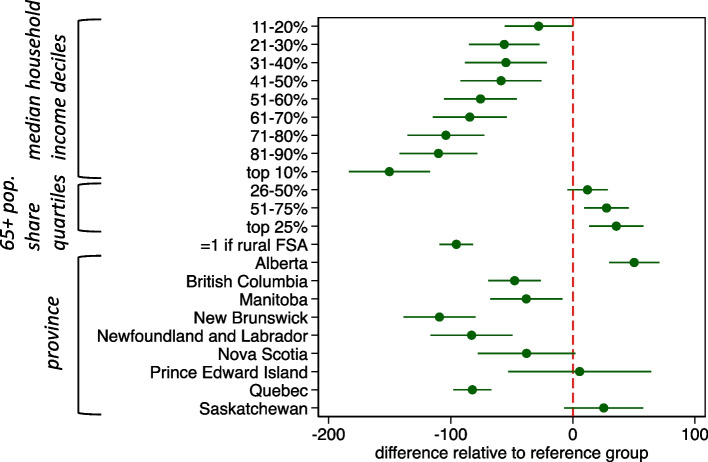
Linear regression of total weekly community pharmacy operating hours per 10,000 FSA population. Note: estimates are the difference in total weekly community pharmacy operating hours per 10,000 population relative to reference group: non-rural Forward Sortation Areas in Ontario, with lowest 10% of median total household income and lowest 25% of elderly population share. Estimated 95% confidence intervals in green

**Fig. 6:**
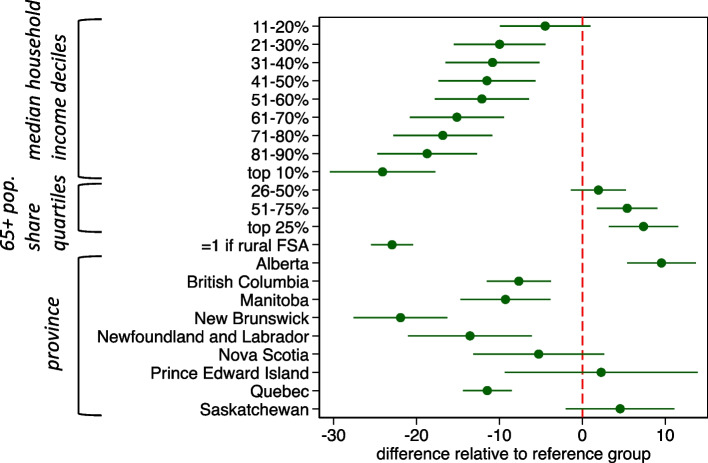
Linear regression of total weekend community pharmacy operating hours per 10,000 FSA population. Note: estimates are the difference in total weekend community pharmacy operating hours per 10,000 population relative to reference group: non-rural Forward Sortation Areas in Ontario, with lowest 10% of median total household income and lowest 25% of elderly population share. Estimated 95% confidence intervals in green

Figure 5 reports the parameter estimates of the model of total FSA level pharmacy weekly operating hours per 10,000 population. The same patterns hold in the models of pharmacy density, but the estimated effects are now larger. For example, there are about 150 fewer operating hours per week per 10,000 population in the most affluent regions compared to the least affluent regions. The interprovincial variation in hours is also substantial, with a 150-h difference between New Brunswick, the province with the fewest hours per 10,000 population, and Alberta, the province with the most. The same patterns are also observed in Fig. 6, which reports parameter estimates of the model of total FSA level pharmacy weekend operating hours per 10,000 population.

The quantile regression estimates are reported in Appendix 2. These results are consistent with the linear regression results shown in Figs. 4, 5 and 6 above.

## Discussion

Pharmacists’ expanded scope of practice raises questions about individuals’ access to pharmacy services. Previous studies for Canada have focussed on the geographic proximity of residential households to pharmacies in Nova Scotia and Ontario. In this paper, I focus on access to pharmacist services across the 10 Canadian provinces using more recent data. I find that pharmacy availability – both the number of pharmacies per capita and the total operating hours of these pharmacies, both overall and on weekends – was higher, the greater the elderly share of the population and the lower is median income.

Given that lower regional income is associated with higher levels of unmet healthcare need, this finding suggests that pharmacies may be well placed to provide care in areas that need it. As an example, earlier research has found that rates of lung cancer and cardiovascular-related mortality are higher in lower income Canadian neighbourhoods [20]. Smoking cessation counselling and hypertension screening – services routinely provided in pharmacies – may be effective in addressing these health problems.

The density of pharmacies in Canada appears to be higher than in the UK, the US and other countries examined in the literature. For instance, Norris and colleagues note that in New Zealand in 2010 there were about 4,800 individuals served per pharmacy; [3] in Canada in 2019 this number was 3,440. There are likely two reasons for the surfeit of pharmacies in Canada. The first reason lies with Canada’s approach to financing prescription drugs, which includes not only drugs themselves, but also payments to pharmacies and wholesalers. The public sector covers less than one half (about 45%) of prescription drug costs, typically for those 65 + , those with very low income and others who face high drug costs relative to income. Voluntary, private insurers cover about 35%; this is mostly employer-sponsored coverage extended to employees and dependents. The remaining 20% is covered directly by patients, typically in the form of copayments and deductibles [21]. The voluntary, private insurance sector is non-existent, or at least much smaller, in other OECD countries [2]. Instead, most OECD countries rely on public drug coverage, or compulsory social insurance coverage, although such coverage usually requires patient cost sharing. This gives the state some control over spending on pharmacist services. The New Zealand government, for instance, sets an annual budget for pharmacist services [22]. Expenditure control is lower in mixed public–private insurance systems, especially systems such as Canada’s where private insurers tend to impose weaker reimbursement constraints than do public plans [23].

While, to the best of my knowledge, there is no literature that compares pharmacy remuneration across countries, it seems to be the case that total pharmacy remuneration is higher in Canada than in other countries examined in the literature. This level of remuneration allows more pharmacies to be commercially viable than otherwise, including pharmacies with lower prescription volumes.

The second possible reason that Canada has a relatively large number of pharmacies is due to its permissive rules on pharmacy ownership and location. All provincial pharmacist regulatory authorities have rules on the books requiring that pharmacies be majority owned by pharmacists. But in most provinces, corporations can work around these rules; [24] pharmacists are still required, however, to manage a corporate-owned pharmacy. Pharmacies in Canada also face no special location restrictions. In many jurisdictions in Europe and elsewhere, new pharmacies must locate a certain distance away from existing pharmacies, or satisfy a local pharmacy needs assessment [25, 26].

The combination of relatively unconstrained pharmacy remuneration in Canada and permissive rules on pharmacy ownership and location help explain Canada’s density of community pharmacies. It is less clear why pharmacies tend to locate in regions with lower household income or higher elderly shares of the population. One possible explanation is that prescription demand (and thus pharmacy sales revenues) is particularly high in these areas. The prevalence of chronic health problems increases with age and those 65 and older are eligible for relatively generous provincial government drug plan coverage. Similarly, there is a well-established association between income and health. But lower income individuals under 65 in Canada typically would not have superior coverage to those with higher income given that they are less likely to qualify for generous employer-sponsored drug coverage. There is public drug coverage for the indigent in each province, and it is possible that lower income individuals who do not qualify for this public coverage are willing to pay out of pocket for pharmacy services. But it is unclear if these are the factors responsible for the income-pharmacy access gradient found in this paper. Further investigation seems warranted.

Our models of regional pharmacy density have limitations. As this analysis relied on observational data, it is possible that there are unmeasured confounders – variables associated with health need factors that independently determine pharmacy availability. However, I was unable to identify any such confounders. Also, individuals who live near a regional border may have access to a pharmacy in a neighbouring FSA, yet this would not be captured. The study is also silent on the distribution of pharmacies *within* an FSA. The previous Canadian studies, however, found that most households in Ontario and Nova Scotia live near a pharmacy. This study focussed on associations between indicators of health needs and availability of pharmacy services. The study, however, does not directly measure health need; different indicators could lead to different results. Finally, this study was silent on the health impacts of regional variations in access to pharmacist services. This remains an area for future research.

## Conclusions

The density of pharmacy services – both the number of pharmacies per capita and the total hours these pharmacies are open – tend to be higher in regions of Canada with older and lower income residential populations. This suggests that pharmacies are well positioned to provide primary care services in these regions.

## Supplementary Information

**Additional file 1: Table A1.1 **Linear regressions of pharmacy availability.

**Additional file 2: Table A2.1 **Quantile regressions of pharmacy availability.

## Data Availability

The datasets used and/or analysed during the current study are available from the corresponding author on reasonable request.

## Abbreviations

ESI: Express Scripts International
FSA: Forward Sortation Area
OECD: Organisation for Economic Co-operation and Development
OLS: Ordinary least squares
