# Supplementary material for: Pharmacy location and medical need: regional evidence from Canada
Source: BMC Health Serv Res. 2022 Nov 3;22:1309. doi: 10.1186/s12913-022-08709-5 (PMC9635116; doi:10.1186/s12913-022-08709-5)
Supplement: Supplementary file 1 — Additional file 1: Table A1.1 Linear regressions of pharmacy availability. [file 12913_2022_8709_MOESM1_ESM.docx]

Table A1.1 Linear regressions of pharmacy availability

m1 = # community pharmacies per 10,000 population.

m2 = total weekly operating hours of community pharmacies per 10,000 population.

m3 = total weekend operating hours of community pharmacies per 10,000 population.

| Variable | m1 | m2 | m3 |
| --- | --- | --- | --- |
| deciles of 2015 FSA median household income |  |  |  |
| 11-20% | -0.47* | -28.02* | -4.48 |
| 21-30% | -0.87*** | -56.19*** | -9.98*** |
| 31-40% | -0.88*** | -54.82** | -10.84*** |
| 41-50% | -0.88** | -58.80*** | -11.52*** |
| 51-60% | -1.23*** | -75.63*** | -12.12*** |
| 61-70% | -1.34*** | -84.42*** | -15.14*** |
| 71-80% | -1.69*** | -104.00*** | -16.84*** |
| 81-90% | -1.81*** | -110.08*** | -18.73*** |
| top 10% | -2.52*** | -150.27*** | -24.11*** |
|  |  |  |  |
| quartiles of share of pop. that is 65+ |  |  |  |
| 26-50% | 0.19 | 11.97 | 1.94 |
| 51-75% | 0.38** | 27.58** | 5.41** |
| top 25% | 0.51** | 35.57** | 7.38*** |
| =1 if rural FSA | -1.02*** | -95.44*** | -22.96*** |
| Alberta | 0.84*** | 50.25*** | 9.54*** |
| British Columbia | -0.66*** | -47.71*** | -7.67*** |
| Manitoba | -0.42 | -38.14* | -9.28*** |
| New Brunswick | -1.55*** | -109.25*** | -21.94*** |
| Newfoundland and Labr.. | -1.39*** | -82.93*** | -13.56*** |
| Nova Scotia | -0.86** | -37.93 | -5.27 |
| Prince Edward Island | -0.11 | 5.58 | 2.27 |
| Quebec | -1.41*** | -82.30*** | -11.47*** |
| Saskatchewan | 0.47 | 25.31 | 4.56 |
| Constant | 4.34*** | 284.58*** | 51.13*** |
| N | 1282 | 1282 | 1282 |
| Adjusted R^2^ | 0.20 | 0.21 | 0.21 |

Legend: * p<0.05; ** p<0.01; *** p<0.001

Table A1.2 Linear regression of # community pharmacies per 10,000 population. FSA average 2019 taxfiler income used instead of 2015 Census median household income.

| Variable |  |
| --- | --- |
| deciles of 2019 FSA average taxfiler income |  |
| 11-20% | -0.51* |
| 21-30% | 0.04 |
| 31-40% | -0.52* |
| 41-50% | -0.69** |
| 51-60% | -0.70** |
| 61-70% | -0.89*** |
| 71-80% | -0.91*** |
| 81-90% | -0.88*** |
| top 10% | -1.25*** |
| quartiles of share of pop. that is 65+ |  |
| 26-50% | 0.51*** |
| 51-75% | 0.92*** |
| top 25% | 1.21*** |
| =1 if rural FSA | -1.02*** |
| Alberta | 0.76*** |
| British Columbia | -0.57** |
| Manitoba | -0.39 |
| New Brunswick | -1.51*** |
| Newfoundland and Labr.. | -1.43*** |
| Nova Scotia | -0.69* |
| Prince Edward Island | -0.08 |
| Quebec | -1.13*** |
| Saskatchewan | 0.63* |
| Constant | 3.32*** |
| N | 1280 |
| Adjusted R^2^ | 0.16 |

Legend: * p<0.05; ** p<0.01; *** p<0.001
