# Supplementary material for: Pharmacy location and medical need: regional evidence from Canada
Source: BMC Health Serv Res. 2022 Nov 3;22:1309. doi: 10.1186/s12913-022-08709-5 (PMC9635116; doi:10.1186/s12913-022-08709-5)
Supplement: Supplementary file 2 — Additional file 2: Table A2.1 Quantile regressions of pharmacy availability. [file 12913_2022_8709_MOESM2_ESM.docx]

Table A2.1 Quantile regressions of pharmacy availability

m1 = # community pharmacies per 10,000 population.

m2 = total weekly operating hours of community pharmacies per 10,000 population.

m3 = total weekend operating hours of community pharmacies per 10,000 population.

| Variable | m1 | m2 | m3 |
| --- | --- | --- | --- |
| deciles of 2015 FSA median household income |  |  |  |
| 11-20% | -0.36 | -7.34 | -4.26 |
| 21-30% | -0.72** | -34.27* | -8.52* |
| 31-40% | -0.76*** | -38.91* | -9.31** |
| 41-50% | -0.79*** | -44.42* | -9.20** |
| 51-60% | -0.81*** | -44.22* | -8.42* |
| 61-70% | -1.07*** | -57.08** | -12.77*** |
| 71-80% | -1.26*** | -69.28*** | -13.24*** |
| 81-90% | -1.37*** | -81.40*** | -14.93*** |
| top 10% | -2.00*** | -112.42*** | -20.63*** |
|  |  |  |  |
| quartiles of share of pop. that is 65+ |  |  |  |
| 26-50% | 0.34* | 19.59 | 2.98 |
| 51-75% | 0.58*** | 39.74** | 6.64** |
| top 25% | 0.72*** | 48.27*** | 10.70*** |
| =1 if rural FSA | -0.74*** | -89.32*** | -23.33*** |
| Alberta | 0.80*** | 58.19*** | 10.79*** |
| British Columbia | -0.56** | -48.98*** | -7.69** |
| Manitoba | -0.23 | -20.53 | -6.66 |
| New Brunswick | -1.21*** | -107.97*** | -27.09*** |
| Newfoundland and Labr.. | -1.00** | -71.42** | -15.72*** |
| Nova Scotia | -0.41 | -19.41 | -3.71 |
| Prince Edward Island | -0.02 | 22.83 | 8.86 |
| Quebec | -1.25*** | -69.71*** | -9.44*** |
| Saskatchewan | 0.71* | 32.33 | 6.41 |
| Constant | 3.75*** | 243.00*** | 46.29*** |
| N | 1282 | 1282 | 1282 |

Legend: * p<0.05; ** p<0.01; *** p<0.001
